# Familiarity Gates Socially Transmitted Aggression via the Medial Amygdala

**DOI:** 10.1523/JNEUROSCI.1018-25.2025

**Published:** 2025-09-08

**Authors:** Magdalene P. Adjei, Elana Qasem, Sophia Aaflaq, Jessica T. Jacobs, Savannah Skinner, Fletcher Summa, Claudia Spotanski, Rylee Thompson, Mikaela L. Aholt, Taylor Lineberry, Jacob C. Nordman

**Affiliations:** Division of Molecular and Integrative Physiology, Department of Biomedical Sciences, Southern Illinois University School of Medicine, Carbondale, Illinois 62903

**Keywords:** familiarity, learned aggression, medial amygdala, social cognition, socially transmitted aggression

## Abstract

Aggressive behavior can be acquired through observation, providing adaptive advantages but also posing significant social risks. In humans, individuals repeatedly exposed to aggression are more likely to engage in violent behavior later in life. Yet, the environmental factors and neural mechanisms underlying observationally acquired aggression remain unclear. Here, we propose that social familiarity with an aggressor is critical for activating neural circuits in observers that primes aggression. To investigate this, we established a novel behavioral paradigm termed “socially transmitted aggression (STA),” in which witness mice observed either familiar or unfamiliar demonstrators attacking intruder mice. Remarkably, male, but not female, witnesses displayed increased aggression only after observing familiar demonstrators, with no effect from unfamiliar ones. Given that excitatory neurons in the posterior–ventral segment of the medial amygdala (MeApv) have been implicated in aggression priming, we hypothesized these neurons might be involved in STA as well. Supporting this hypothesis, fiber photometry revealed selective activation of excitatory MeApv neurons during familiar, but not unfamiliar, demonstrator attacks. Chemogenetically and optogenetically inhibiting these neurons suppressed STA, while activating them during unfamiliar demonstrator attacks promoted aggression. These results establish social familiarity as essential for the observational transmission of aggression and identify excitatory MeApv neurons as critical mediators of this phenomenon, offering potential avenues for clinical intervention.

## Significance Statement

Observational learning of aggression contributes significantly to antisocial behavior, yet the neural circuits and social factors underlying this phenomenon remain poorly understood. This study reveals that familiarity with an aggressor is essential for socially transmitted aggression in mice, mediated by activation of excitatory neurons in the posterior–ventral medial amygdala (MeApv). Using fiber photometry, chemogenetics, and optogenetics, we demonstrate that MeApv neuronal activity is necessary and sufficient for the expression of learned aggression. These findings uncover a neural circuit mechanism linking social identity with aggressive behavior, highlighting a potential therapeutic target for reducing pathological aggression and violence transmitted within familiar social groups.

## Introduction

Aggression is a complex social behavior with deep evolutionary roots, capable of conferring both adaptive advantages and serious liabilities. While direct aggressive encounters have been studied extensively, it has become increasingly clear that aggression can also be acquired by observing the aggressive behavior of others.

Classic work in children by Bandura and colleagues showed that brief exposure to an aggressive model or regular exposure to film-mediated aggression escalates later violence (Bandura et al., 1961, 1963a,b), and longitudinal studies link repeated observation of family or peer aggression to delinquency and criminal acts (Bandura et al., 1961, 1963a,b; Dodge and Coie, 1987; Lansford et al., 2007). Translational animal research echoes this pattern, demonstrating that mice will attack more after watching conspecific fights (Iravedra-Garcia et al., 2024). This observational form of aggression carries significant implications for public health and social development. Despite clear behavioral parallels across species, the environmental filters and neural circuits that convert observed aggression into future offensive action remain poorly defined.

Social-learning paradigms in rodents suggest that the identity of the demonstrator strongly shapes what the observer learns. Rats copy food choices only from cage-mates, not strangers (Galef and Wigmore, 1983; Heyes, 1994), and the intensity of observational fear rises when demonstrator and observer are familiar (Bruchey et al., 2010; Jeon et al., 2010). Whether a similar rule applies to aggression is unresolved. Two recent mouse studies illustrate this ambiguity. Yang et al. found hypothalamic aggression “mirror” neurons that fired during active and observed fighting (Yang et al., 2023). However, these observers did not increase their own attacks, possibly because the demonstrators were unfamiliar. In contrast, Iravedra-Garcia et al. reported heightened observer aggression but only after 10 daily observation sessions, a schedule that likely fostered emergent familiarity (Iravedra-Garcia et al., 2024). Together, these studies suggest that social familiarity may be a critical gate that translates witnessed aggression into active aggression.

The medial amygdala (MeA) decodes pheromonal and social cues relevant to threat, fear, and attack (Hong et al., 2014; Falkner et al., 2020). We previously found that excitatory (CaMKIIα^+^/vGlut2^+^) neurons within the posterior–ventral subdivision of the MeA (MeApv) drive aggression priming—a transient escalation of violence after a single fight (Nordman et al., 2020a). Because both aggression priming and socially learned aggression depend on attack experience, it is plausible these excitatory MeApv neurons are similarly activated during observed aggression, priming mice when the aggressor is familiar. This is supported by the fact that the MeApv densely projects onto the ventrolateral subdivision of the ventromedial hypothalamus (VmHvl) where the hypothalamic aggression “mirror” neurons reside (Nordman et al., 2020a,b; Bartsch et al., 2023; Yang et al., 2023; Iravedra-Garcia et al., 2024). In this conception, the primary role of the MeApv in observed aggression is to provide crucially important information about the identity of the aggressor necessary to guide behavioral outcomes.

Therefore, we hypothesize that social familiarity gates the transmission of aggressive behavior via the activation of excitatory MeApv neurons. To test this hypothesis, we created a two-stage “socially transmitted aggression” (STA) assay. Here, a witness CD-1 mouse watches a demonstrator (either familiar or unfamiliar) interact with an intruder across a perforated divider. Thirty minutes later, the witness undergoes its own intruder-interaction test. To determine the role of the MeApv in STA, we combined fiber photometry with chemogenetic and optogenetic manipulations to monitor, inhibit, and then activate MeApv neurons while observing demonstrator aggression. This strategy allowed us to determine whether familiarity gates STA, if the excitatory MeApv neurons of witnesses are preferentially activated when observing a familiar attacking demonstrator, and whether activation of the MeApv is necessary and sufficient for STA.

This study will aid in determining how violence propagates through tightly knit social groups and highlights the MeApv as a potential therapeutic target for disorders marked by pathological aggression.

## Materials and Methods

### Animals

All animal protocols were approved by the Animal Care and Use Committee of Southern Illinois University School of Medicine. All CD1 and C57BL/6 mice used in this study were purchased from Charles River Laboratories, culled prior to arrival in our animal facility, and then group-housed with their litter mates in a 19.37 × 18.06 × 39.83 cm polycarbonate cage (Allentown) on a reverse 12 h light/dark cycle (lights off 8:30 A.M.–8:30 P.M.) with *ad libitum* access to water and food. We refer to the group-housed mice as familiar if they had persistent and unrestricted tactile and olfactory contact from birth (indicated by Charles River Laboratories). Unfamiliar animals were characterized as having no previous interaction prior to testing. CD1 mice were exclusively used as witnesses and demonstrators, while C57 mice were only used as intruders. Surgical procedures, outlined below, were performed at 5–6 weeks of age, and then the mice were group-housed for an additional 3–4 weeks. All behavioral testing was conducted at the same age of 8–9 weeks.

### STA paradigm

**Figure 1.**
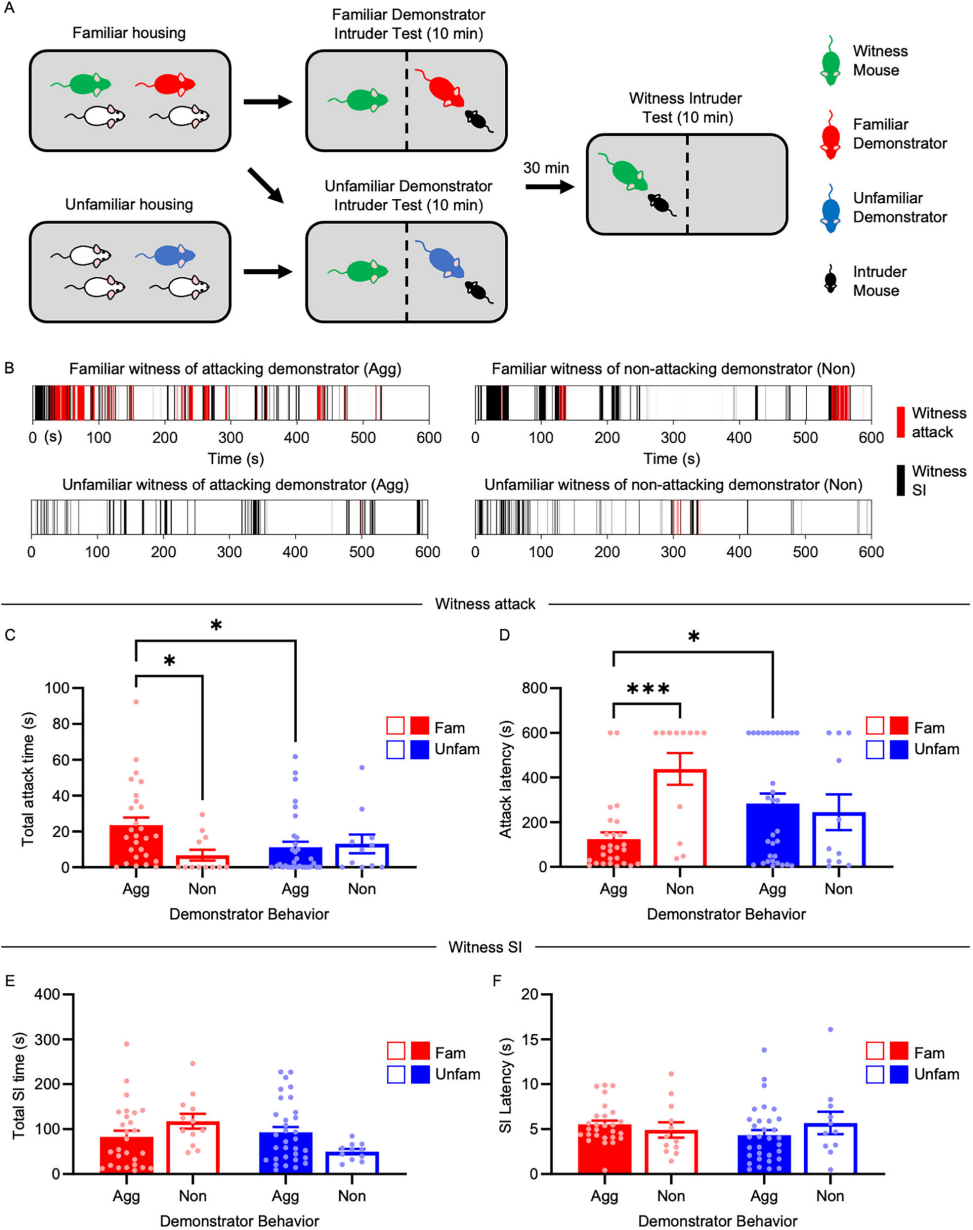
STA depends on familiarity. ***A***, Social transmission paradigm (see Materials and Methods and text for details). Witness mice that did not observe a demonstrator attack were used as controls. ***B***, Representative raster plots of witness behavior during the witness intruder test. ***C–F***, Quantification of total attack time (***C***), attack latency (***D***), total SI time (***E***), and SI latency (***F***) during the witness intruder test (*n* = 27, 13, 32, 12). A latency of 600 s indicates that the mouse did not attack at any point during the recording session. Mice exposed to aggressive demonstrators are depicted with closed bars (Agg) and mice exposed to non-aggressive demonstrators are depicted with open bars (Non) Data are mean ± SEM. *p < 0.05; ***p < 0.001.

Group-housed 8–9-week-old mice were transferred to a darkened behavior room and left alone to acclimate for at least 1 h prior to aggression testing. Familiar or unfamiliar CD1 mice were then positioned on opposite sides of a clear perforated divider in a novel, high-walled cage, lit by an overhead light, and left to acclimate for an additional 10 min (Fig. 1*A*). We elected to use a novel cage to reduce the effect of territoriality on aggression (Koolhaas et al., 2013) and a clear, perforated divider and overhead light because previous studies have shown that demonstrator-induced activation of aggression circuits require visual cues (Yang et al., 2023). Tactile contact between the two mice was not allowed during testing to avoid disruption.

A novel mouse (C57) was then introduced into one side of the arena, and the two mice were allowed to *ad libitum* interact for 10 min while the mouse on the other side of the divider observed. We refer to the observing mouse as the “witness” and the paired CD1 mouse as the “demonstrator.” We also refer to this portion of the test as the “demonstrator–intruder test.” After the 10 min demonstrator–intruder test, the demonstrator and C57 mouse were removed and returned to their home cages. After a 30 min wait period, a new intruder (C57) was introduced into the witness side, and the two mice were allowed to *ad libitum* interact for 10 min, which we refer to as the “witness–intruder test.” All mice (demonstrators, witnesses, and intruders) were only used once. Animal behavior was captured via a tripod-mounted video camera. If excessive tissue damage occurred, the test was prematurely terminated and not analyzed (*n* = 2 mice). Behavior videos were reviewed and manually scored using BehaviorCloud by a researcher blind to the experimental conditions. Aggressive behaviors are operationally defined as biting the rear, biting the front/face, chasing, and wrestling (Blanchard and Blanchard, 1977; Koolhaas et al., 2013; Nordman et al., 2020a, 2022). Nonaggressive social behavior is defined as anogenital sniffing, nonaggressive investigation, and flank rubbing.

Female mice, which were only used for the experiments presented in Figure 1 and Figure S1, did not display aggression against female intruders during the demonstrator or witness intruder tests regardless of familiarity (*n* = 8 for each condition, 0% attacked). Therefore, only male mice were used for the remainder of the study. Estrous was not determined prior to testing.

### Surgical procedures

All procedures were performed on 5–6-week-old CD1 male mice that were anesthetized with isoflurane (3% for induction and 1–2% for maintenance) and then placed onto a stereotaxic frame (David Kopf Instruments) for viral injection and fiber implantation. To restrict expression of our transgenes to excitatory neurons in the MeApv, all viral constructs were driven by the CaMKIIα promoter, which we previously showed colocalizes almost exclusively with type 2 vesicular glutamate transporter (vGlut2)+ MeApv neurons, indicating they are glutamatergic (Nordman et al., 2020a,b; Bartsch et al., 2023; Mojahed et al., 2024).

For chemogenetic experiments, a bilateral craniotomy was made, and 400 nl of pAAV9-CaMKIIα-hM4D(Gi)-mCherry (hM4Di, Addgene #50477, 9.9 × 10^12^ vg/ml), pAAV9-CaMKIIα-hM3D(Gq)-mCherry (hM3Dq, Addgene #50476, 2.5 × 10^13^ vg/ml), or pAAV9-CaMKIIα-mCherry control (mCherry, Addgene #114469, 2.1 × 10^13^ vg/ml) was injected into each MeApv (AP, −1.5 mm; ML, ±2.1 mm; DV, −5.25 mm) using a 5 μl gas-tight Neuros Hamilton Syringe coupled to a 33 gauge stainless steel needle ejected at a rate of 40 nl/min by a syringe pump (World Precision Instruments). After injection, the syringe was left in place for an additional 10 min and then slowly withdrawn. The skin was sealed using Vetbond. Ketoprofen (5 mg/kg) was administered for 3 d postsurgery. Mice were allowed to recover for 3–4 weeks before STA testing.

For fiber photometry and optogenetic experiments, we unilaterally injected 250 nl of AAV9-CaMKIIα-GCaMP6f.WPRE.SV40 (GCaMP6f, Addgene #100834, 2.3 × 10^13^ vg/ml) or bilaterally injected 300 nl of pAAV9-CaMKIIα-eNpHR 3.0-EYFP [enhanced halorhodopsin (eNpHR), Addgene #26971, 2.2 × 10^13^ vg/ml] into the MeApv of 5–6-week-old male CD1 mice at a rate of 40 nl/min, respectively. Control mice were injected with pAAV9-CaMKIIα-YFP (YFP, Addgene #26971, 2.2 × 10^13^ vg/ml) at the same rate. After viral injection, fiber optical ferrules (photometry, diameter 1.25 mm and depth 5.5 mm, black; optogenetics, diameter 2.5 mm and depth 5.5 mm, white; RWD Life Science) were implanted 0.1 mm above the MeApv. The fibers were secured to the skull with stainless steel screws (1.6 mm, Pro-Tech International), a thin layer of Metabond (Parkell), and then a larger layer of acrylic dental cement (Lang Dental). Once the cement had fully cured, animals were placed back in their home cage on a preheated pad at 37°C. Ketoprofen was administered for 3 d postsurgery. Mice were allowed to recover for 3–4 weeks before STA testing.

### Fiber photometry recording

Calcium activity was recorded exclusively from the male witnesses during the demonstrator–intruder phase of the STA assay using a tricolor fiber-photometry system (R820, RWD Life Science). Although the system houses 410, 470, and 560 nm LEDs, only the 410 nm (isosbestic reference) and 470 nm (GCaMP6f excitation) channels were employed. Both LEDs were routed through a dual-band 410/470 nm dichroic and a 200 µm core, 0.37 NA patch cord to a chronically implanted ferrule (RWD Life Science) positioned 0.1 mm above the MeApv. Excitation power at the ferrule tip was set to 20 µW (Thorlabs photodiode meter) to minimize bleaching while maintaining signal-to-noise. Fluorescence returned through the same fiber and was captured by the system's integrated sCMOS detector at 60 Hz; synchronized behavioral video was acquired simultaneously at 30 fps (640 × 480 px).

Motion artifacts and bleaching were corrected by fitting the 410 nm trace to the 470 nm trace with least squares regression and computing Δ*F*/*F* = (470 nm − fitted 410 nm) / fitted 410 nm. The resulting trace was *z*-scored to a 2 s pre-event (attack or nonaggressive social behavior) baseline for each bout. The area under the curve (AUC) values for the 6 s postevent window were compared with baseline AUCs using two-tailed paired *t* tests with Bonferroni’s adjustments; bouts containing other events within the baseline window were omitted automatically by custom MATLAB scripts. Recordings were excluded if histology revealed fiber misplacement or off target viral expression (*n* = 2).

### Brain sectioning and image acquisition for viral confirmation

Mice were transcardially perfused with 4% paraformaldehyde (PFA) in phosphate-buffered saline (PBS) solution. Brains were removed and postfixed at 4°C overnight and then cryoprotected overnight in 15% sucrose (in PBS) followed by 30% sucrose (in PBS). Brains were cut into 40-μm-thick sections using a cryostat (Leica CM3050-S) and then mounted onto charged slides (Thermo Fisher Scientific) with Vectashield HardSet Antifade Mounting Medium containing DAPI. To confirm location of viral injection, we imaged the sections with a fluorescent microscope (EVOS) using a 10× (NA 0.45) objective. Any animals showing viral expression or fiber placement outside of the MeApv were excluded from analysis.

### Foot shock

Eight-to-nine-week-old virally injected (hM4Di, eNpHR, GFP, or hM3Dq) male mice were transferred from their housing room to a behavior room shielded from light and then allowed to acclimate for 1 h before being placed into a white light illuminated fear conditioning chamber within a sound-attenuating cubicle (Med Associates). After a 3 min exploration period, three electric shocks (0.7 mA, 1 s in duration) were administered through an electrified grate at random intervals of 240–480 s over 15 min. Mice were then returned to their home cage for 30 min before being killed for immunohistochemistry experiments.

### Immunohistochemistry

Male mice expressing hM4Di, eNpHR, GFP, or hM3Dq were transcardially perfused with 4% PFA in PBS 30 min after footshock. Brains were extracted, postfixed in 4% PFA overnight at 4°C, and cryoprotected sequentially in 15% and then 30% sucrose in PBS. The tissue was sectioned at 30 μm using a cryostat (Leica CM3050-S) and stored as free-floating sections in PBS for immunohistochemistry. Sections were blocked for 2 h at room temperature in PBS-T (0.03% Triton X-100) containing 10% goat serum and 1% bovine serum albumin. They were then incubated overnight at 4°C with a primary antibody against c-Fos (1:2,000; Abcam, #ab190289), followed by a 1 h incubation at room temperature with either Alexa Fluor 488- or 555-conjugated secondary antibodies (1:200; Thermo Fisher Scientific #A-11034 or #A-21428). Finally, sections were mounted onto slides with Vectashield HardSet Antifade Mounting Medium containing DAPI (Vector Laboratories).

### Chemogenetic experiments

On the test day, surgical animals (all males) were transferred from their housing room to a darkened behavior room and left alone to acclimate for 1 h prior to aggression tests. Witness mice were injected with 0.9% saline (vehicle) or 2 mg/kg clozapine N-oxide (CNO, Sigma-Aldrich), the effective dose in our previous studies (Bartsch et al., 2023; Mojahed et al., 2024), 30 min before the demonstrator–intruder test. The remainder of the STA assay was conducted as described above. Manipulations were excluded if histology revealed off target viral expression (*n* = 3).

### Optogenetic recording

Neuronal activity in the MeApv of males was suppressed using the inhibitory opsin, eNpHR. Bilateral optical fibers (200 µm core, 0.37 NA; 0.1 mm above MeApv) were connected to a laser (Opto Engine) delivering continuous yellow light (590 nm, 10 mW measured at the ferrule tip) through a patch cord for the entire 10 min demonstrator–intruder test. This stimulation window with eNpHR has been successfully deployed to suppress neural activity and alter behavior (Carter et al., 2010). Control mice expressing YFP underwent the identical light-delivery protocol. No mice showed viral expression outside of the MeApv or fibers positioned other than above the MeApv.

### Experimental design and statistical analyses

The GraphPad Prism software was used for statistical analysis. All analyses were blind to condition. Power analyses for each experiment were calculated using G*power and adjusted based on previous studies (Nordman et al., 2020a). All analyses assume a standard deviation of 20%, 1-*β* of 0.8, and an *α* of 0.05. Assumptions were checked for all experimental models using the Shapiro–Wilk test of normality and Levene's test for equality of variance. *P* < 0.05 was considered significant, and all tests were two-tailed. All data were presented as individual data points or expressed as mean ± SEM. Details can be found in Table S1.

## Results

### STA depends on familiarity

We began by examining whether aggression could be transmitted through observation and the role of familiarity in this process. To test this, we developed a paradigm where familiar or unfamiliar group-housed CD1 mice are positioned on opposite sides of a transparent perforated divider in a novel arena exposed to an overhead light (Fig. 1*A*). We selected CD1 mice as they have been shown to be particularly adept at social learning (Lee et al., 2025). As described in the methods section, we used a novel cage to reduce the effect of territoriality on aggression (Koolhaas et al., 2013) and a clear perforated divider and overhead light due to the requirement of visual cues for activation of aggression circuitry (Yang et al., 2023).

During testing, a C57 intruder is placed into the one side of the arena, and the two mice are allowed to *ad libitum* interact for 10 min, while the mouse on the other side of the divider observes. We refer to the observing mouse as the “witness” and the paired CD1 mouse as the “demonstrator.” We refer to this portion of the STA assay as the “demonstrator–intruder test.” The intruder and demonstrator are then removed and placed into their respective cages. We previously showed that attack experience leads to a short-term escalation in aggression, known as aggression priming, which is most effective after a 30 min wait period. Therefore, we elected to use this same wait period in the current study (Nordman et al., 2020a). After the 30 min wait period, a new C57 is placed into the witness side, and the two are allowed to ad libitum interact for 10 min, consistent with our aggression-priming protocol. We refer to this portion of the test as the “witness intruder test.”

Our results show that male witnesses become more aggressive when observing a familiar, but not unfamiliar, attacking demonstrator [interaction effect (familiarity × demonstrator attack): total attack time, *F*_(1,78)_ = 4.773; *p* = 0.0319; Fig. 1*C*; attack latency, *F*_(1,78)_ = 9.946; *p* = 0.0023; Fig. 1*D*,*B*; Table S1]. Familiar witnesses were also nearly three times as likely to attack if they observed an aggressive demonstrator than a nonattacking demonstrator (92.6% from an attacking demonstrator, 33.3% from a nonattacking demonstrator; Fig. S1*A*). Importantly, familiarity did not affect demonstrator aggression (Fig. S1*B*,*C*; Table S1) or witness aggression if the demonstrator did not attack (Fig. 1*B–D*; Table S1). Neither familiar or unfamiliar female demonstrators or witnesses attacked female intruders under these conditions (*n* = 8 for each condition, 0% attacked); therefore, subsequent analyses focused on males.

Having established that familiarity gates STA, we next asked whether the variability in witness aggression could be attributed to how vigorously a demonstrator fought. Among familiar pairs, demonstrator attack time significantly correlated with witness aggression (total attack time, *r*^2^ = 0.1447; *p* = 0.0169; Fig. S1*D*; attack latency, *r*^2^ = 0.3862; *p* < 0.0001; Fig. S1*E*; Table S1). No significant correlations were found for unfamiliar pairs (total attack time, *r*^2^ = 0.0004050; *p* = 0.8981; Fig. S1*F*; attack latency, *r*^2^ = 0.007239; *p* = 0.5875; Fig. S1*G*; Table S1). These findings indicate that among familiar pairs, demonstrator attack intensity reliably predicts witness attack intensity.

Our previous study showed that aggression priming had no effect on nonaggressive social behavior (SI; Nordman et al., 2020a), and so we assessed whether exposure to an attacking or nonattacking demonstrator could affect SI as well. We found an interaction effect for total SI time (*F*_(1,78)_ = 6.239; *p* = 0.0146; Fig. 1*B*,*E*; Table S1), but no main effects or significant differences between groups after post hoc analysis (Fig. 1*B*,*E*; Table S1). There were no significant differences in SI latency between groups (Fig. 1*B*,*F*; Table S1). These results indicate that exposure to demonstrator SI has minimal effects on witness SI.

Together, these results indicate that observing the attack behavior of a familiar mouse can promote aggression in the witness, suggesting that aggression can be primed through social modeling.

### STA activates excitatory neurons in the MeApv

Our previous study demonstrated that aggression priming depends on the activation of excitatory MeApv neurons (Nordman et al., 2020a). Therefore, we hypothesized that the excitatory MeApv neurons of witnesses are activated when observing a familiar demonstrator attack, underlying STA. To test this, we performed calcium imaging using fiber photometry. Fiber photometry is a live imaging technique that captures the population activity of specific neurons using fluorescent reporters such as the calcium indicator GCaMP (Dana et al., 2019). We chose fiber photometry over traditional methods such as c-Fos staining due to its capability to capture real-time fluctuations in genetically defined neuronal populations, which can be precisely aligned with specific behaviors—essential for our STA paradigm. Additionally, as demonstrated in Figure S1*D*–*G*, witness aggression intensity strongly correlates with demonstrator aggression within familiar pairs. This implies variability in MeApv neuron activation levels dependent on demonstrator behavior, which could introduce substantial variability in c-Fos quantification. Fiber photometry effectively mitigates this issue by providing real-time activity measures linked directly to behavioral events.

**Figure 2.**
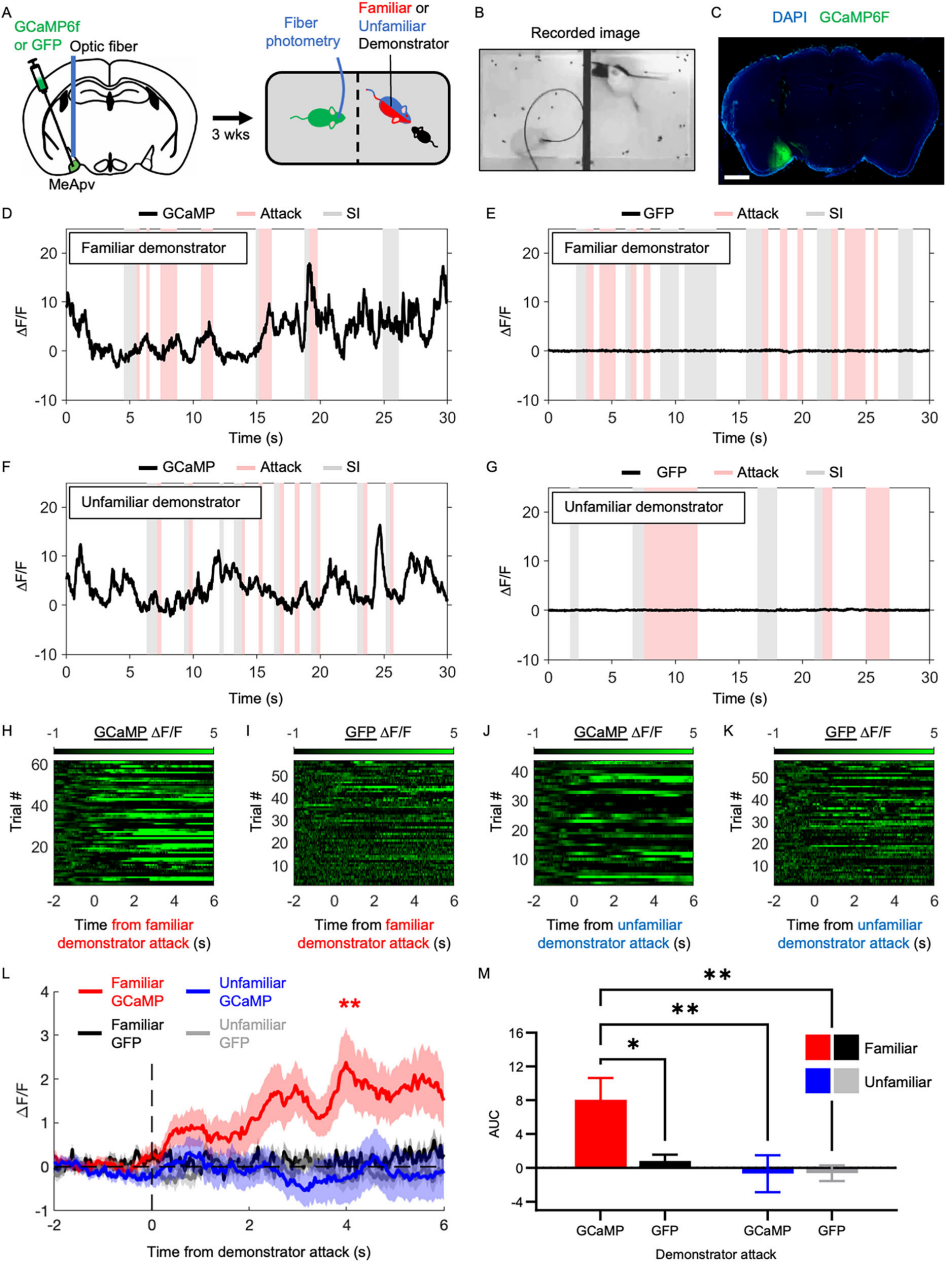
Familiar demonstrator attack activates the excitatory MeApv neurons of witnesses. ***A***, Experimental procedure. Mice were unilaterally injected with GCaMP6F or GFP control virus into the MeApv and implanted with an optic fiber 0.1 mm above. Three weeks later, MeApv activity of witnesses was measured during the demonstrator–intruder test. Red and blue mice refer to familiar and unfamiliar demonstrators, respectively. ***B***, Still of a video recorded photometry session, showing a demonstrator mid-attack (white mouse in the right compartment of the arena). ***C***, Representative image of GCaMP6F expression in the MeApv (scale bar, 1 mm). ***D–K***, Graphs and raster plots showing fluorescence changes in the MeApv of GCaMP (***D***, ***F***, ***H***, ***J***) or GFP (***E***, ***G***, ***I***, ***K***) expressing witnesses observing demonstrator attack. ***L***, Average change in the fluorescence signal of GCaMP- or GFP-expressing witnesses observing familiar or unfamiliar demonstrator attack (*n* = 62, 57, 44, 58). Colored lines indicate group averages and shaded areas indicate SEM. ***M***, Bar graph comparing the AUC of demonstrator attack-evoked responses (0–6 s) from GCaMP or GFP-expressing witnesses (*n* = 62, 57, 44, 58). Data are mean ± SEM. *p < 0.05; **p < 0.01.

Adeno-associated virus (AAV) encoding the fast, ultra-bright calcium indicator GCaMP6f or GFP (under the control of the CaMKIIα promoter) was injected into the MeApv of 5–6-week-old CD1 mice, which were then implanted with an optical fiber 0.1 mm above (Fig. 2*A*). A representative still of the recording assay and image of GCaMP6f expression in the MeApv can be found in Figure 2, *B* and *C*, respectively. The spread of the virus in the MeA for each animal is shown in Figure S2*A*.

After 2-to-3 weeks, we recorded the activity of the excitatory MeApv neurons of witnesses during the demonstrator–intruder test (Fig. 2*B*,*D*–*M*; Fig. S2). We found a significant increase in the calcium signal of witnesses observing a familiar, but not unfamiliar, attacking demonstrator (familiar demonstrator attack, *t* = 1.9996; *p* = 0.0029; Fig. 2*D*,*H*,*L*; unfamiliar demonstrator attack, *t* = 2.0167; *p* = 0.8288; Fig. 2*F*,*J*,*L*; Table S1). No change was found in GFP controls (familiar, *t* = 2.0032; *p* = 0.3446; Fig. 2*E*,*I*,*L*; unfamiliar, *t* = 2.0025; *p* = 0.5368; Fig. 2*G*,*K*,*L*; Table S1). Consistent with this data, two-way ANOVA and post hoc analysis revealed that GCaMP-expressing familiar witnesses showed significantly stronger attack-evoked responses compared with unfamiliar witnesses and GFP controls (*F*_(1,217)_ = 4.026; *p* = 0.0416; Fig. 2*M*; Table S1).

Interestingly, we also found a significant increase in the calcium signal of witnesses observing familiar, but not unfamiliar, SI (familiar, *t* = 2.0032; *p* = 0.0007; Fig. S2*A*,*E*; unfamiliar, *t* = 2.0227; *p* = 0.2622; Fig. S3*C*,*E*; Table S1). Fluorescence changes were not observed in GFP controls (familiar, *t* = 2.0129; *p* = 0.3876; Fig. 2*E*; Fig. S3*B*,*E*; unfamiliar, *t* = 2.0117; *p* = 0.8387; Fig. 2*G*; Fig. S3*D*,*E*; Table S1). When comparing SI-evoked responses between the four groups, two-way ANOVA showed no interaction effect (*F*_(1,217)_ = 1.458; *p* = 0.2216; Fig. S3*F*; Table S1), and post hoc analysis revealed no difference between GCaMP-expressing familiar and unfamiliar mice. However, there was a main effect of viral expression (*F*_(1,217)_ = 8.808; *p* = 0.0034; Fig. S3*F*; Table S1), and post hoc analysis revealed significant differences between GCaMP-expressing familiar witnesses and GFP controls.

One potential explanation for the calcium increase after SI is that having observed attacks throughout the familiar demonstrator–intruder test, the MeApv neurons were now activated by any interaction that had the potential of turning antagonistic. In support of this, familiar witnesses observing no attack during the demonstrator–intruder test showed no significant change in MeApv activity during SI, similar to GFP controls (GCaMP, *t* = 2.0301; *p* = 0.5335; GFP, *t* = 2.0860; *p* = 0.1703; Fig. S3*G*–*I*; Table S1). SI-evoked responses were also not significantly different between GCaMP- and GFP-expressing mice (*t* = 0.2007; *p* = 0.8416; Fig. S3*L*; Table S1). These data strongly suggest that SI in the absence of anticipated aggression does not significantly activate MeApv neurons.

These results indicate that MeApv neurons are activated in witnesses observing a familiar, but not unfamiliar, attacking demonstrator, suggesting a key role in STA.

### Chemogenetic inhibition of excitatory MeApv neurons reduces STA

**Figure 3.**
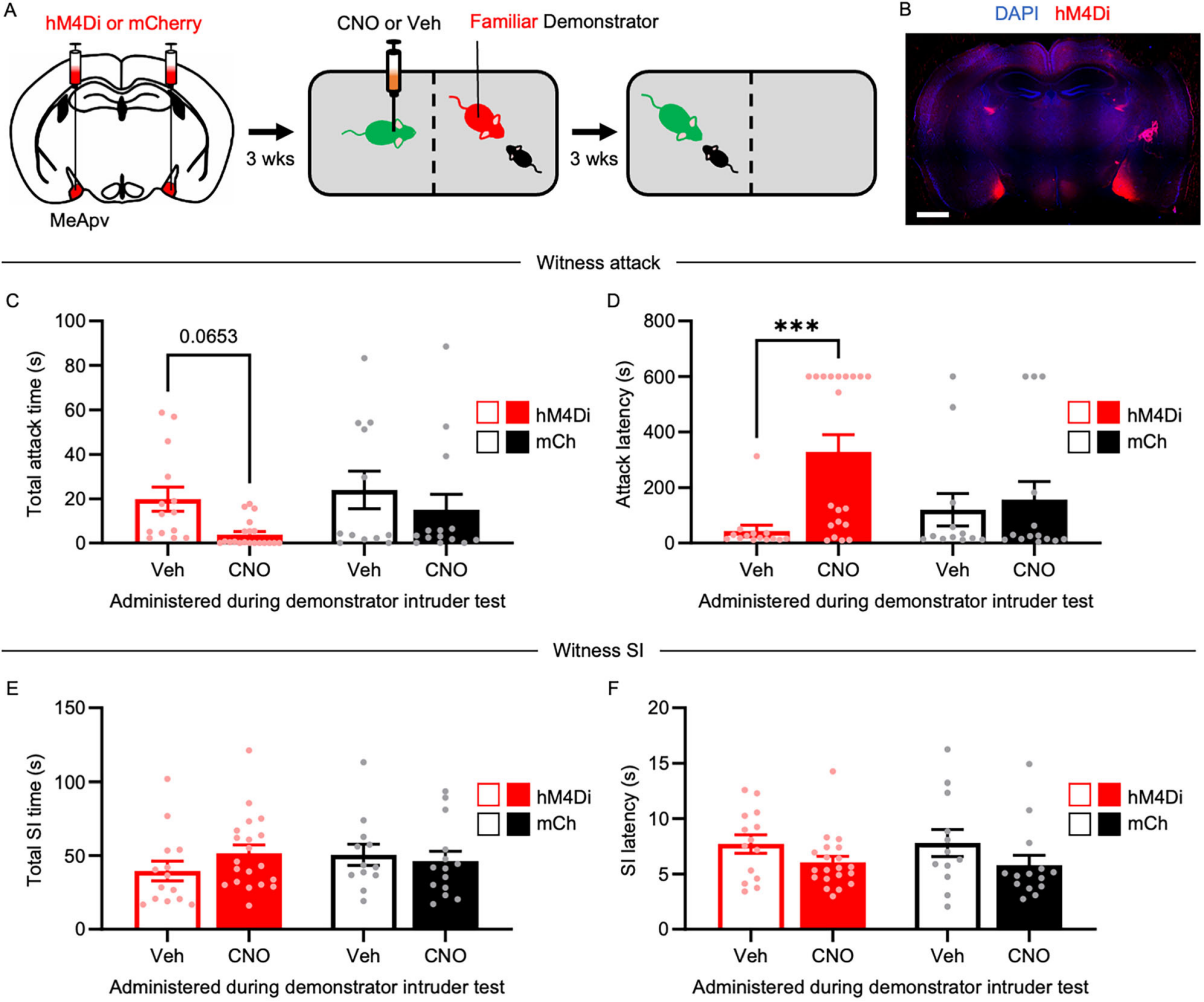
Chemogenetic inhibition of excitatory MeApv neurons reduces STA in familiar witnesses. ***A***, Experimental schedule. Mice were bilaterally injected with hM4Di or mCherry into the MeApv, followed 3 weeks later by intraperitoneal injections of 2 mg/kg CNO or Veh 30 min before the familiar demonstrator–intruder test. ***B***, Representative image of hM4Di expression (red) in the MeApv (scale bar, 1 mm). ***C–F***, Quantification of total attack time (***B***), attack latency (***C***), Total SI time (***D***), and SI latency (***E***) during the witness intruder test (*n* = 14, 20, 12, 14). Data are mean ± SEM. **p < 0.001.

To assess the role of excitatory MeApv neurons in familiarity-dependent STA, we used a chemogenetic approach. hM4Di or mCherry control virus was injected into the MeApv of 5–6-week-old mice which were then group-housed for an additional 3–4 weeks (Fig. 3*A*). hM4Di is an inhibitory designer receptors exclusively activated by designer drug (DREADD) receptor that chemogenetically silences neurons (Roth, 2016).

For these experiments, we used familiar pairs of mice to promote STA. We began by administering an intraperitoneal injection of the DREADD-selective ligand CNO (2 mg/kg) or vehicle to witnesses 30 min before performing the familiar demonstrator–intruder test. We have successfully used CNO to suppress MeApv activity related to traumatic stress-induced aggression (Bartsch et al., 2023; Mojahed et al., 2024) and have confirmed this finding in Figure S4*A*–*C* (c-Fos/hM4Di, *t* = 3.783; *p* = 0.0054; Table S1). The experimental design and a representative image of hM4Di expression in the MeApv are displayed in Figure 3, *A* and *B*. The spread of the virus in the MeA for each animal is shown in Figure S2*B*.

During the witness intruder test, two-way ANOVA showed an interaction effect of drug treatment and virus on attack latency, but not total attack time (total attack time, *F*_(1,56)_ = 0.4019; *p* = 0.5287; Fig. 3*C*; attack latency, *F*_(1,56)_ = 4.577; *p* = 0.0368; Fig. 3*D*; Table S1). However, there was a main effect of drug treatment on both total attack time and attack latency (total attack time, *F*_(1,56)_ = 5.117; *p* = 0.0276; Fig. 3*C*; attack latency, *F*_(1,56)_ = 7.671; *p* = 0.0076; Fig. 3*D*; Table S1), showing that CNO treatment in hM4Di-expressing witnesses produced a significantly longer attack latency and a trend toward lower total attack time compared with their vehicle-injected counterparts (total attack time, *p* = 0.0653; Fig. 3*C*; attack latency, *p* = 0.001; Fig. 3*D*). Post hoc analyses showed no effect of CNO on mCherry controls (Fig. 3*C*,*D*). As before, we also assessed SI in witnesses and found no differences between conditions (Fig. 3*E*,*F*; Table S1). Together, these results indicate that excitatory MeApv neurons regulate STA.

### Optogenetic inhibition of excitatory MeApv neurons attenuates STA

**Figure 4.**
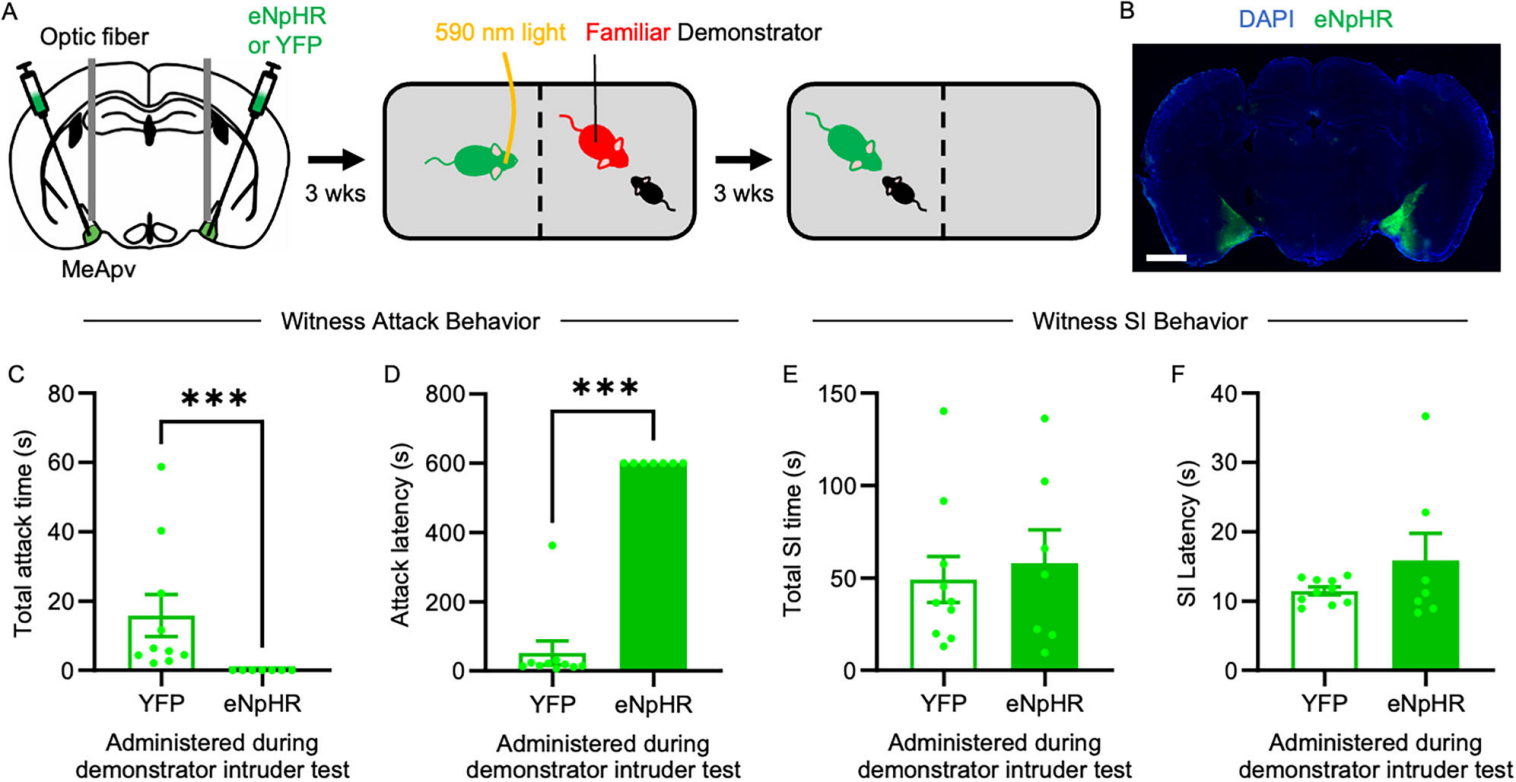
Optogenetic inhibition of excitatory MeApv neurons reduces STA in familiar witnesses. ***A***, Experimental schedule. Mice were bilaterally injected with eNpHR or GFP control virus into the MeApv and implanted with optical fibers 0.1 mm above. Three weeks later, witnesses were photostimulated with 590 nm light during the familiar demonstrator–intruder test. ***B***, A representative image of eNpHR expression in the MeApv (scale bar, 1 mm). ***C–F***, Quantification of total attack time (***C***), attack latency (***D***), total SI time (***E***), and SI latency (***F***) during the witness–intruder test (*n* = 10, 7). Data are mean ± SEM. ***p < 0.001.

To achieve precise temporal control over MeApv neural activity in witnesses during demonstrator attack, we used an optogenetic approach. AAV encoding the inhibitory opsin, eNpHR, or YFP (again under the control of the CaMKIIα promoter) was bilaterally injected into the MeApv of 5–6-week-old mice, and then optical fibers were implanted 0.1 mm above (Fig. 4*A*,*B*). This approach allows us to directly test whether MeApv activity during the witnessing phase is required for the subsequent expression of STA. The spread of the virus in the MeApv for each animal is shown in Figure S2*C*. Confirmation of our ability to suppress MeApv neural activity using eNpHR can be found in Figure S4*D*–*F* (c-Fos/GFP or eNpHR, *t* = 5.025; *p* = 0.0007; Table S1).

Inhibiting the MeApv neurons of witnesses during the familiar demonstrator–intruder test almost completely abolished STA compared with YFP-expressing controls (total attack time, *U* = 0; *p* < 0.0001; Fig. 4*C*; attack latency, *U* = 0; *p* < 0.0001; Fig. 4*D*; Table S1). Importantly, SI was not affected by MeApv inhibition (total SI time, *U* = 32; *p* = 0.8125; Fig. 4*E*; attack; SI latency, *U* = 35; *p* > 0.9999; Fig. 4*F*; Table S1), consistent with our chemogenetic manipulations and further confirming that the MeApv selectively regulates STA without disrupting SI. These findings demonstrate that activation of the MeApv is necessary for the expression of STA.

### Chemogenetic activation of excitatory MeApv neurons promotes STA between unfamiliar pairs of mice

**Figure 5.**
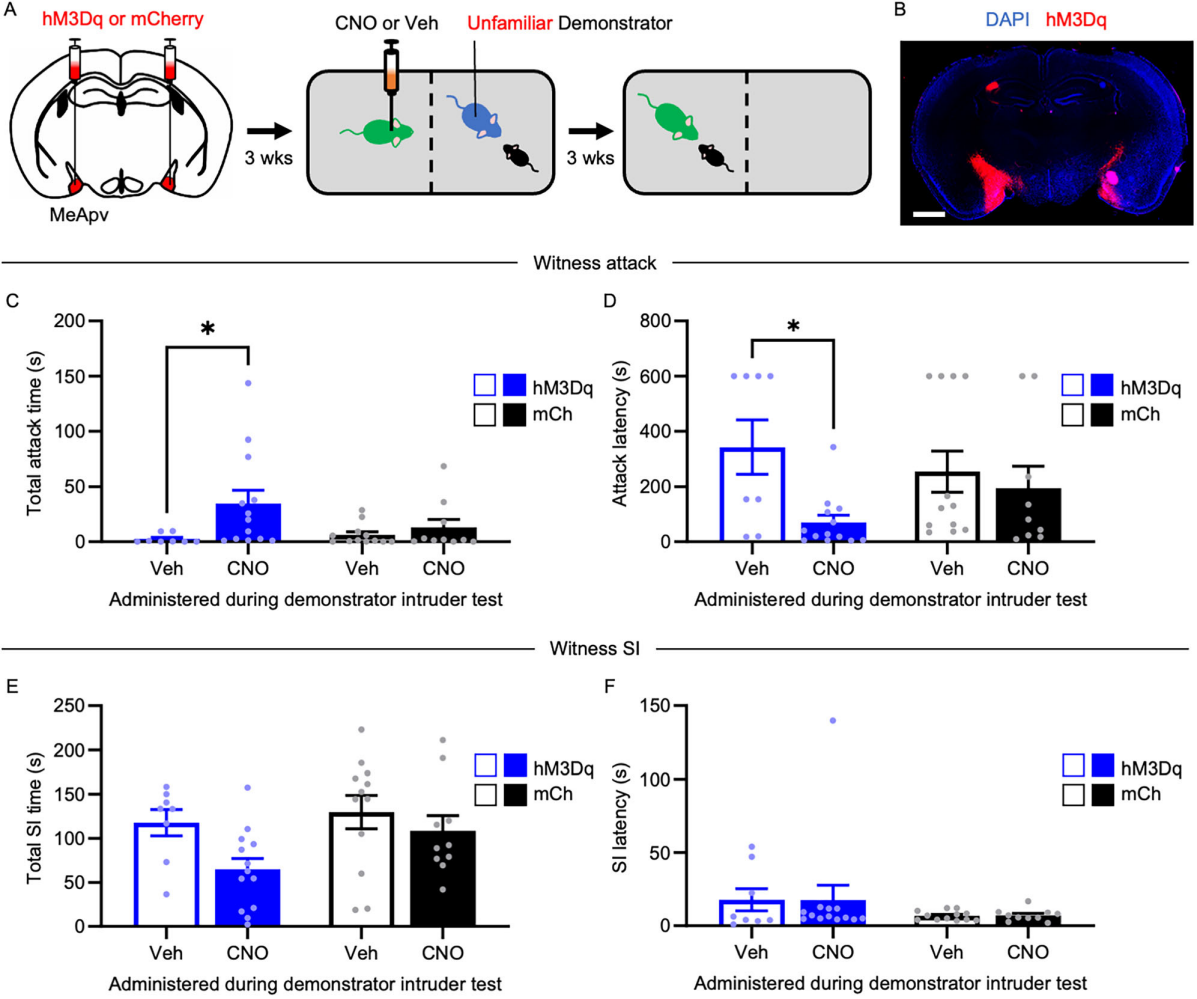
Chemogenetic activation of excitatory MeApv neurons promotes STA in unfamiliar witnesses. ***A***, Experimental schedule. Mice were bilaterally injected with hM3Dq or mCherry into the MeApv, followed 3 weeks later by intraperitoneal injections of 2 mg/kg CNO or Veh 30 min before the unfamiliar demonstrator–intruder test. ***B***, Representative image of hM3Dq expression (red) in the MeApv (scale bar, 1 mm). ***D–F***, Quantification of total attack time (***C***), attack latency (***D***), total SI time (***E***), and SI latency (***F***) during the witness intruder test (*n* = 8, 13, 12, 10). Data are mean ± SEM. *p < 0.05.

Finally, we tested to see if chemogenetically activating the excitatory MeApv neurons during unfamiliar demonstrator attack could promote STA. Here, we expressed the activating DREAAD receptor hM3Dq or mCherry control (under control of the CaMKIIα promoter) into the MeApv of 5–6-week-old mice and then behaviorally tested the mice in our STA paradigm 3 weeks later (Fig. 5*A*,*B*). The spread of the virus in the MeApv for each animal is shown in Figure S2*D*. Confirmation of our ability to activate the MeApv using CNO can be found in Figure S4*G*–*I* (c-Fos/hM3Dq, *t* = 6.697; *p* < 0.0001; Table S1).

On the test day, we administered intraperitoneal injections of 2 mg/kg CNO or vehicle to witnesses 30 min before the unfamiliar demonstrator–intruder test. Unfamiliar pairs were used as they do not produce STA and so start at low-baseline levels of witness aggression. While interaction effects were not found (total attack time, *F*_(1,39)_ = 2.202; *p* = 0.5287; Fig. 5*C*; attack latency, *F*_(1,39)_ = 2.339; *p* = 0.1345; Fig. 5*D*; Table S1), there was a main effect of drug treatment on attack metrics (total attack time, *F*_(1,39)_ = 5.246; *p* = 0.0276; Fig. 5*C*; attack latency, *F*_(1,39)_ = 5.117; *p* = 0.0299; Fig. 5*D*; Table S1). Post hoc analysis supported that CNO-injected hM3Dq–expressing witnesses attacked more than the vehicle-injected witnesses (total attack time, *p* = 0.0256; Fig. 5*C*; attack latency, *p* = 0.0179; Fig. 5*D*). As with our previous manipulations, there was no significant difference in SI between conditions (Fig. 5*E*,*F*; Table S1).

These findings suggest that activating the MeApv can largely simulate familiarity during the unfamiliar demonstrator–intruder test, driving STA. Taken together, this study demonstrates that the MeApv is a critical nucleus for STA.

## Discussion

This study identifies social familiarity and MeApv activity as key determinants of STA, a newly characterized form of learned aggression in mice. Using a novel behavioral paradigm alongside fiber photometry, chemogenetics, and optogenetics, we show that witnessing aggression by a familiar conspecific—but not an unfamiliar one—primes later aggressive behavior, but not SI (Fig. 1). This effect is facilitated by selective activation of excitatory MeApv neurons in familiar witnesses (Fig. 2). Causal manipulations confirm necessity and sufficiency: chemogenetic or optogenetic silencing of these neurons during the observation phase abolishes STA (Figs. 3, 4), whereas chemogenetic activation induces STA even between unfamiliar pairs (Fig. 5) while largely sparing SI. Together, these findings position the MeApv as a subcortical hub that integrates social identity with observed aggression to gate future aggressive behavior, providing a mechanistic entry point for understanding how violence propagates through cohesive social groups.

### Familiarity in STA

Our findings identify familiarity as a key gating mechanism for STA. Mice only exhibited aggression after witnessing a familiar demonstrator attack, suggesting that social identity strongly influences whether observed aggression is encoded and later reproduced. This aligns with studies showing that familiarity modulates social learning and emotional contagion in rodents. For example, rats mimic food choices from cage-mates, but not strangers (Galef and Wigmore, 1983; Heyes, 1994), and observational fear is enhanced when observers are familiar with the demonstrator (Bruchey et al., 2010; Jeon et al., 2010).

These results also help reconcile conflicting findings in the observational aggression literature. For example, one study reported activation of hypothalamic “mirror neurons” during observed aggression, but no behavioral transmission—likely due to the use of unfamiliar demonstrators (Yang et al., 2023). Another study found that repeated observation of an unfamiliar attacking demonstrator led to increased aggression, possibly because familiarity developed over time (Iravedra-Garcia et al., 2024). By directly manipulating social familiarity, our study demonstrates that this factor is both necessary and sufficient to gate STA.

### STA as experience-dependent aggression

Our findings suggest that STA shares mechanistic features with other forms of experience-dependent aggression, including aggression priming and traumatic stress-induced aggression. All three involve recent exposure to aggression-related events—whether direct (as in priming), stressful (as in trauma), or observed (as in STA)—that lead to enhanced aggressive behavior during future social encounters. A common thread across these models is the MeApv, which appears to serve as a convergence node for encoding socially or emotionally salient experiences.

In previous work, we showed that aggression priming depends on NMDAR-dependent synaptic potentiation at glutamatergic MeApv-VmHvl synapses (Nordman et al., 2020b). Traumatic stress likewise induces long-lasting aggression through the strengthening and addition of these same connections (Nordman et al., 2020a,b; Bartsch et al., 2023; Mojahed et al., 2024). The current results extend this framework to observational learning: witnessing familiar aggression activates MeApv neurons and induces a behavioral phenotype consistent with priming, suggesting that similar plasticity mechanisms with the VmHvl may be engaged. Whether observed aggression can directly potentiate MeApv-VmHvl synapses remains an open question, but fiber photometry and manipulation experiments suggest that MeApv circuits are both necessary and sufficient for STA expression.

This convergence raises the possibility that STA represents a form of vicarious priming: a mechanism by which aggression can spread socially, not just through direct experience but through observation of violence among familiar peers. Indeed, vicarious behavioral priming has been seen in stress paradigms where animals observing social defeat display increased depressive-like and anxiety-like behaviors and drug and alcohol reward sensitivity (Ródenas-González et al., 2023). Our findings suggest this could extend to aggression, contributing to a cycle wherein violence is socially transmitted within tightly connected groups.

However, while our results demonstrate that a single exposure to familiar aggression is sufficient to activate MeApv neurons and transiently prime aggressive behavior, the mechanisms by which such exposure could lead to more persistent behavioral changes remains to be fully defined. In real-world settings, individuals often witness violence repeatedly within familiar social groups, a pattern that may more strongly engage long-term plasticity mechanisms. This is corroborated by studies showing that repeatedly winning fights can induce synaptic and structural plasticity between the posterior amygdala (PA) and VmHvl to produce prolonged increases in attack behavior (Stagkourakis et al., 2020; Yan et al., 2024). Given the anatomical and functional convergence between aggression priming, repeated fighting contests, traumatic stress-induced aggression, and STA, we propose that repeated MeApv or PA activation via social observation may similarly induce lasting synaptic changes that stabilize aggression-priming circuits. This could provide a mechanistic basis for how vicarious exposure to violence—particularly among familiar individuals—leads to enduring increases in aggressive behavior. Future work will test whether repeated STA experiences elicit structural remodeling of MeApv or PA outputs and whether such changes predict long-term aggression escalation.

### Limitations and future directions

Several caveats temper the generality of our conclusions. First, we were unable to elicit STA in females. Whether this reflects a true sex difference or simply low-baseline aggression of the strain (CD1) remains unclear; testing more aggressive female lines [e.g., Swiss Webster (Newman et al., 2019)] will determine how broadly STA applies across sex and genotype. Estrous was also not considered, but could have played a role in female STA. However, we have previously shown that, regardless of estrous, female mice do not undergo aggression priming (data not shown), suggesting that female mice would be unaffected by estrous in STA as well. Second, all neural data were collected with population-level fiber photometry and bulk chemogenetic/optogenetic manipulations. These approaches cannot reveal whether the same MeApv ensemble supports STA, aggression priming, and stress-induced aggression, therefore leaving open whether “aggression-priming mirror neurons” exist. Cell-resolved calcium imaging using a minimicroscope in combination with the targeted recombination in active populations (TRAP2) system (DeNardo et al., 2019) could be used to address this question; however, the minimicroscope is bulky and likely not suitable for group-housing. Therefore, photometry was the preferred method for this study.

A further limitation of our study is that the chemogenetic activation experiment (Fig. 5) does not allow us to fully discriminate the role of the MeApv in STA from naturally occurring aggression. Continuous activation during the unfamiliar demonstrator–intruder test could inadvertently enhance baseline aggression independently of STA. Indeed, we and others have previously shown that activation of the MeApv can drive naturally occurring and primed aggression (Nordman et al., 2020a,b; Abellan-Alvaro et al., 2022; Qu et al., 2024). Nevertheless, our chemogenetic approach demonstrates the relevance of MeApv activation to aggression priming in the context of STA. Future experiments will clarify the precise timing required for MeApv activation to specifically drive STA.

Finally, we did not evaluate the role of social dominance in STA, yet rank could shape how aggressive behavior is socially acquired. Dominant mice engage more readily in social interaction and are unusually sensitive to social stressors such as isolation (Matthews et al., 2016). Consequently, hierarchy could bias the interpretation of an aggressive demonstration: a dominant witness might treat a familiar conspecific's victory as reinforcing and later escalate its own aggression, whereas a subordinate witness might focus on distress cues and suppress aggression or shift to alternative stress-related behaviors. Addressing this possibility will require establishing rank before testing—using, for example, the tube-test assay (Zhou et al., 2017)—and then stratifying STA expression by dominance status. Such experiments would reveal whether social hierarchy is a critical individual-difference variable that gates the transmission of aggression.

## Conclusion

Collectively, our results reveal STA as an experience-dependent, familiarity-gated phenomenon that engages MeApv neurons previously shown to be involved in direct fighting. Demonstrating that activation of MeApv neurons is both necessary and sufficient for this form of observational escalation in aggression pinpoints a subcortical hub through which social experience can recalibrate later aggression. Clarifying this mechanism lays the groundwork for strategies to blunt the lasting behavioral repercussions of violent exposure.
